# Implant migration and functional outcome of Reverse Shoulder Lateralized Glenosphere Line Extension System: a study protocol for a randomized controlled trial

**DOI:** 10.1186/s13063-022-06482-8

**Published:** 2022-07-19

**Authors:** Marie Louise Jensen, Bo S. Olsen, Marc R. K. Nyring, Müjgan Yilmaz, Michael M. Petersen, Gunnar Flivik, Jeppe V. Rasmussen

**Affiliations:** 1 Department of Orthopedic Surgery, Herlev and Gentofte Hospital, Copenhagen University Hospital, Copenhagen, Denmark; 2 Department of Orthopedic Surgery, Rigshospitalet, Copenhagen University Hospital, Copenhagen, Denmark; 3 Department of Clinical Medicine, University of Copenhagen, Copenhagen, Denmark; 4 Department of Orthopedic Surgery, Skaane University Hospital, Lund University, Lund, Sweden

**Keywords:** Rotator cuff arthropathy, Reverse shoulder arthroplasty, Lateralized glenosphere, Scapular notching, Radiostereometric analysis, Clinical outcome

## Abstract

**Background:**

Inferior scapular notching is a complication unique to reverse shoulder arthroplasty. The most efficient technique to avoid inferior scapular notching has been reported to be lateralization of the glenoid offset. This study aims to compare radiological and functional outcomes of the DELTA Xtend® Reverse Shoulder System Lateralized Glenosphere Line Extension (intervention group) with the Standard DELTA Xtend® Reverse Shoulder System (control group). We hypothesize that the lateralization improves the patient outcome by decreasing the risk of inferior scapular notching without increasing the risk of migration and loosening of glenoid component.

**Methods:**

In this randomized controlled trial, all Danish citizens with rotator cuff arthropathy or degeneration of the glenohumeral joint with severe posterior wear and allocated for a reverse total shoulder arthroplasty at the department of orthopaedic surgery at Herlev and Gentofte Hospital, Copenhagen University Hospital, will be considered for participation. The exclusion criteria are as follows: below 50 years of age, cognitive or linguistic impairment, insufficient glenoid bone stock, previous fracture in the upper extremity and autoimmune-mediated inflammatory arthritis. There will be included a total of 122 patients of which 56 will participate in the radiostereometric analysis. This number of patients allows 20% to drop out. The co-primary outcomes are the pattern and magnitude of the migration of the glenoid component assessed by radiostereometric analysis and the Western Ontario Osteoarthritis of the Shoulder index. The secondary outcomes are inferior scapular notching, patient-reported and functional outcomes (Oxford shoulder score, Constant-Murley score and pain), side effects and complications, changes in bone mineral density and economy. The included patients will be examined before the surgery, within 1 week and 3, 6, 12 and 24 months after.

**Discussion:**

No previous studies have compared the conventional reverse shoulder arthroplasty with the lateralized reverse shoulder arthroplasty in a randomized controlled trial regarding migration and functional outcome. Furthermore, radiostereometric analysis has not been used to evaluate the migration of reverse shoulder arthroplasty in a randomized controlled trial. This study intends to determine which treatment has the most optimal outcome for the benefit of future patients with an indication for reverse shoulder arthroplasty.

**Trial registration:**

The study has been notified to Pactius and has approval number P-2021-231. Furthermore, the study will be registered on Clinicaltrials.gov before starting the inclusion.

**Supplementary Information:**

The online version contains supplementary material available at 10.1186/s13063-022-06482-8.

## Background

Reverse total shoulder arthroplasty is the preferred implant for patients with degeneration of the glenohumeral joint and wear or tear of at least one rotator cuff tendon [1, 2], and the aim of the surgery is pain relief, improved range of motion and relatively low risk of revision surgery [3, 4].

Inferior scapular notching, which is damage to the inferior part of the bone of the glenoid component, is a complication unique to reverse shoulder arthroplasty and was described and graded by Sirveaux et al. in 2004 [4] and reported that 63% of the patients showed signs of inferior scapular notching. A more recent study [5] reported inferior scapular notching in 10% of 476 reverse shoulder arthroplasty implants and found lower clinical outcome scores, lower strength and range of motion (ROM) when present. Both studies found higher revision rates in this subgroup of patients [4, 5]. Furthermore, Roche et al. [6] reported that inferior scapular notching may decrease glenoid baseplate stability which might ultimately lead to aseptic loosening in the clinical situation.

Different techniques to avoid inferior scapular notching have been reported [7]. Lawrence et al [8] reported that the most efficient technique was to use a lateral offset of the glenoid component. Previous studies have found that a lateralized design of the reverse shoulder arthroplasty led to improved rotation and a lower risk of scapular notching [9, 10].

Theoretically, the lateralized design can be associated with a greater risk of loosening of the glenoid component due to the tilt forces [9, 10]. Migration and loosening of the glenoid component can have severe consequences, not only for the patients but also for healthcare providers. Thus, even few extra revisions can make the lateralized component less attractive from an economic point of view.

Plain x-rays are unable to detect minor implant migration and authors have recommended that radiostereometric analysis should be used instead [11, 12]. By inserting small tantalum beads into the surrounding bone, implant migration can be measured extremely accurate [13]. Technical advances within radiostereometric analysis have made it possible to identify the implant and its position using the geometry of the implant instead of attaching tantalum beads to the implant (model-based radiostereometric analysis).

In shoulder arthroplasty surgery, radiostereometric analysis has been used to study the migration of the glenoid component in anatomical total shoulder arthroplasty [14, 15] and, in a few cases, migration of hydroxy-coated resurfacing humeral components [16, 17]. Previous studies have used radiostereometric analysis to access migration of the reverse shoulder arthroplasty [18, 19]. To our knowledge, conventional reverse shoulder arthroplasty and lateralized reverse shoulder arthroplasty systems have not been compared in a randomized design regarding the clinical outcome and patient-reported outcome.

This randomized controlled trial aims to compare the DELTA Xtend® Reverse Shoulder Lateralized Glenosphere Line Extension System (intervention group) to the Standard Delta Xtend® Reverse Shoulder System (control group) (Depuy Synthes, Raynham, MA, USA). We hypothesize that the lateralization improves the patient outcome by decreasing the risk of inferior scapular notching without increasing the risk of migration and loosening of glenoid component.

## Methods and design

### Study design

This study is an investigator initiated, single-centre, 1:1 randomized controlled trial, which will compare the DELTA Xtend® Reverse Shoulder Lateralized Glenosphere Line Extension (intervention group) with the standard DELTA Xtend® Reverse Shoulder System (control group). The two implants are produced by Depuy Synthes (Raynham, MA, USA). The SPIRIT reporting guidelines [20] and checklist (Additional file 1: Appendix 1) were used.

### Method

Inclusion criteria:

1. Rotator cuff arthropathy defined as degeneration of the glenohumeral joint and wear or tear of at least one rotator cuff tendon
2. Degeneration of the glenohumeral joint with intact rotator cuff function but severe posterior wear of the glenoid (> 20° posterior wear)
3. Insufficient effect of non-surgical treatment with symptoms severe enough to justify shoulder arthroplasty.
4. ASA (American Society of Anesthesiology) score 1–3, physically fit for surgery and rehabilitation

**Fig. 1:**
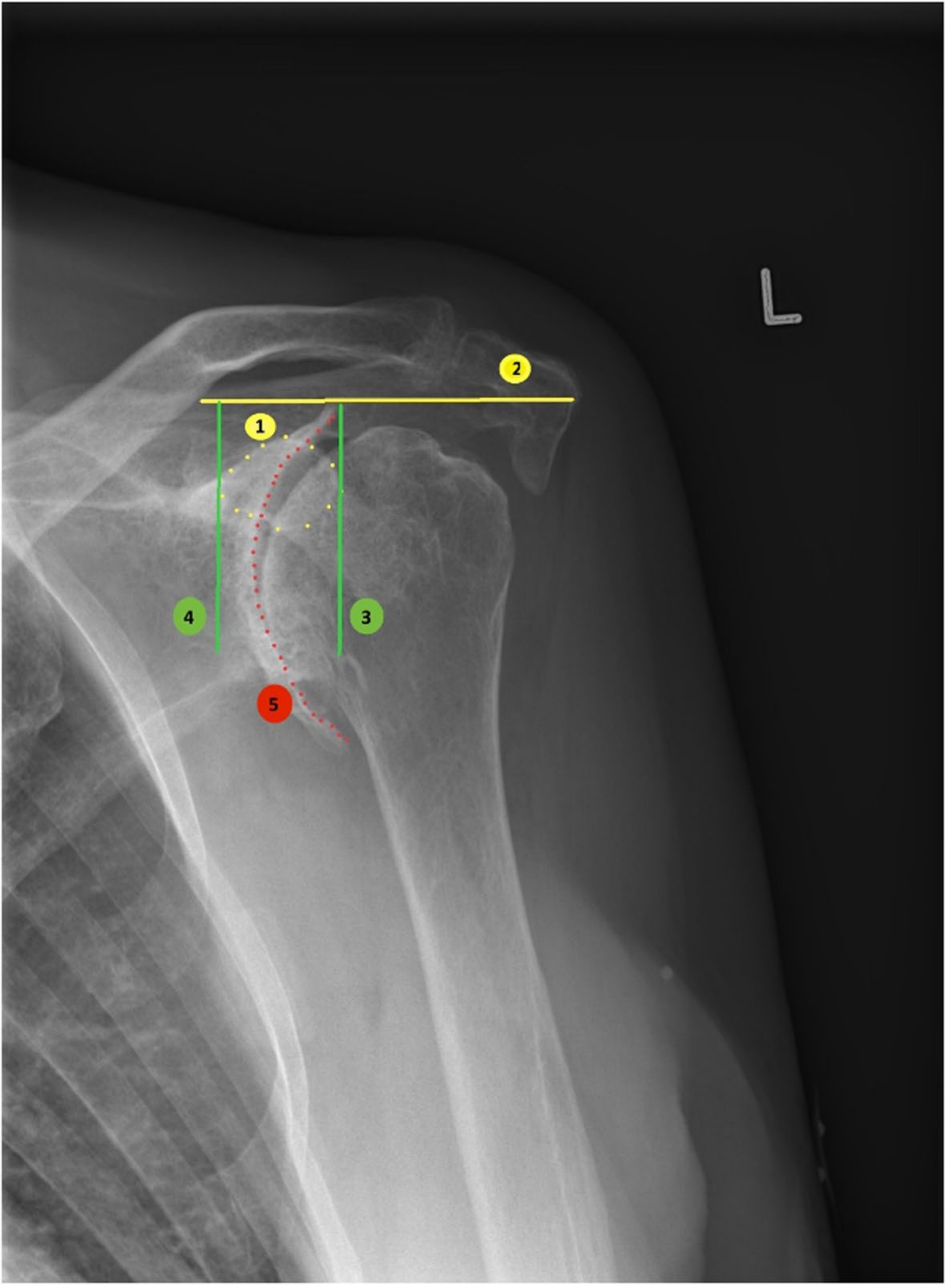
Assessment of medialization on true AP radiograph

Exclusion criteria:

1. Below 50 years of age
2. Cognitive or linguistic impairment
3. Insufficient glenoid bone-stock
4. Previous fracture in the upper extremities
5. Patients with autoimmune-mediated inflammatory arthritis
6. Glenoid border medial to the medial border of the coracoid on a true AP radiograph (Fig. 1)

### Enrolment

**Fig. 2:**
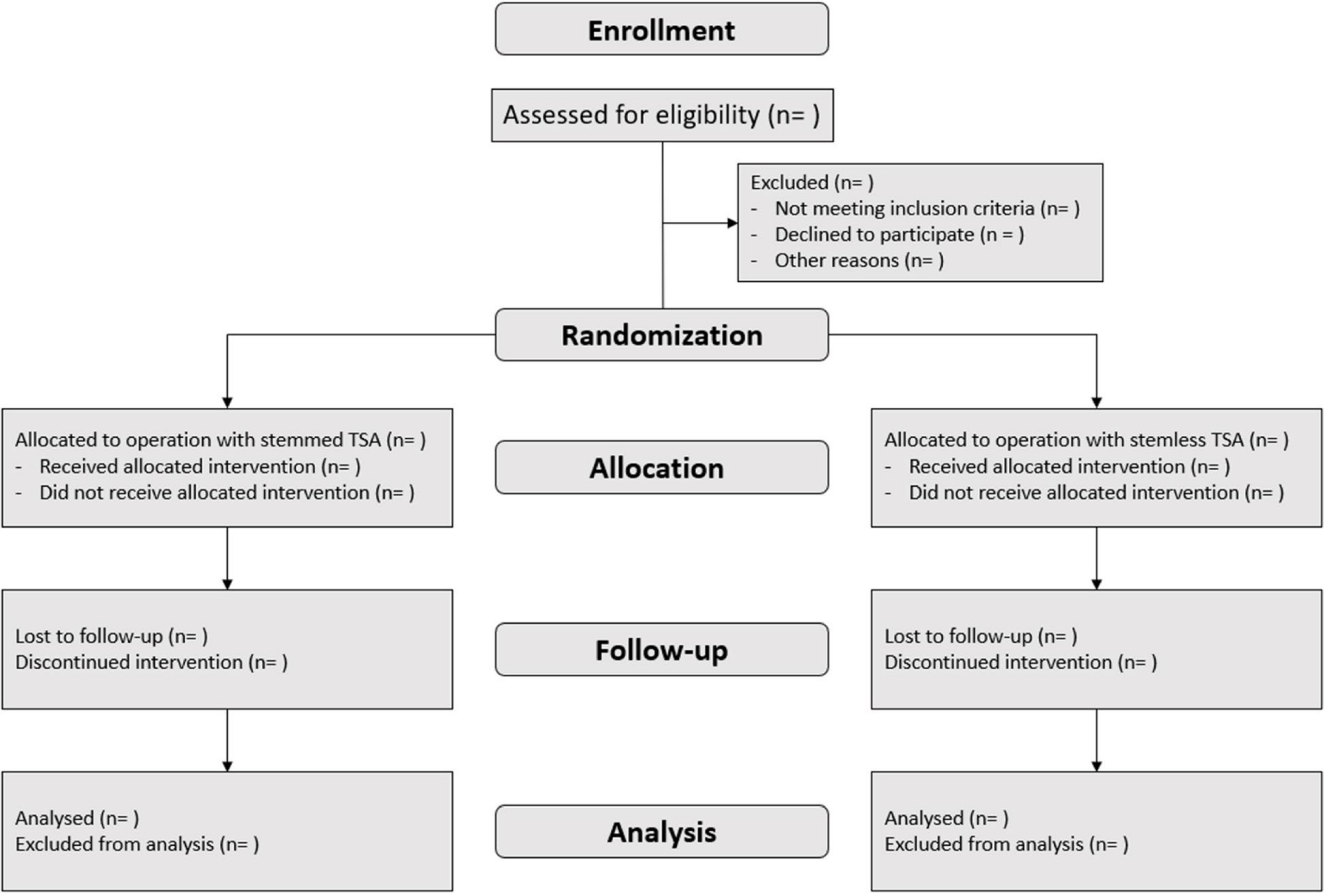
CONSORT flow diagram of the progress of the study

All Danish citizens with the diagnosis of rotator cuff arthropathy (degeneration of the glenohumeral joint and wear or tear of at least one rotator cuff tendon) or degeneration of the glenohumeral joint with severe posterior wear with an indication of a reverse total shoulder arthroplasty referred to the orthopaedic department at Herlev and Gentofte Hospital, Copenhagen University Hospital, will be considered for participation in the trial. Elective surgery is only performed on patients with a Danish civil registration number, which is given at birth or immigration. Thus, no foreign citizens will be included in the trial. The medical records will be reviewed by the treating surgeon, who will evaluate if the patient can participate in the study, based on the above-mentioned inclusion and exclusion criteria. The patients will not be included in the study if the glenoid surface is medial to the medial border of the coracoid process on a true AP x-ray. The measurement is done by the following steps on true AP x-ray: (1) the coracoid process is identified; (2) a reference line is drawn horizontal on the acromion; (3) a perpendicular line is drawn to the acromion reference line, passing through the lateral side of the coracoid process base; (4) a perpendicular line is drawn to the acromion reference line passing through the medial side of the coracoid process base; and (5) the glenoid base is identified (Fig. 1). If the criteria are fulfilled, the treating surgeon will pass information (Additional files 2 and 3: Appendices 2 and 3) to the primary investigator (Fig. 2), and participation will be offered to the patient. The patients who are interested in participation are invited for an undisturbed consultation with the treating surgeon. If the surgeon determines the patient to be eligible, the patient will receive written and oral information about the project. Additionally, the patient will be informed of the possibility to bring a witness for the last consultation before surgery, which is the time point where the surgery is planned and where the patient gives informed consent. This consultation held with the primary investigator, is minimum 24 h later than the previous consultation, minimum 24 h prior to the surgery and in an undisturbed room. The patient and a possible witness will be re-informed of the study and the informed consent (Additional file 4: Appendix 4) will be collected if the patient accepts to participate. Thereby the patient has had minimum 24 h to reflect on the decision and the opportunity to bring a witness.

The patients will then be asked to complete four questionnaires (Oxford Shoulder Score (OSS), Western Ontario Osteoarthritis of the Shoulder index (WOOS score), EQ- 5D-5 L and pain with the visual analogue scale (VAS-score)). The informed consent gives the primary investigator access to information regarding medication, occupation, age, gender, comorbidity and education, from medical records and questionnaires. Furthermore, the patients will have an examination with measurement of the activity of daily living, pain, strength (Constant-Murley score), range of movement and a radiographic examination with plain radiographs with standard anterior-posterior and lateral projections, MRI of the index shoulder joint and CT scan. The glenoid medialization will be measured by using true AP radiograph (Fig. 1). Dual-energy x-ray absorptiometry (DXA) is used to assess bone mineral density (BMD) and osteopenia of the proximal humerus and distal forearm.

**Fig. 3:**
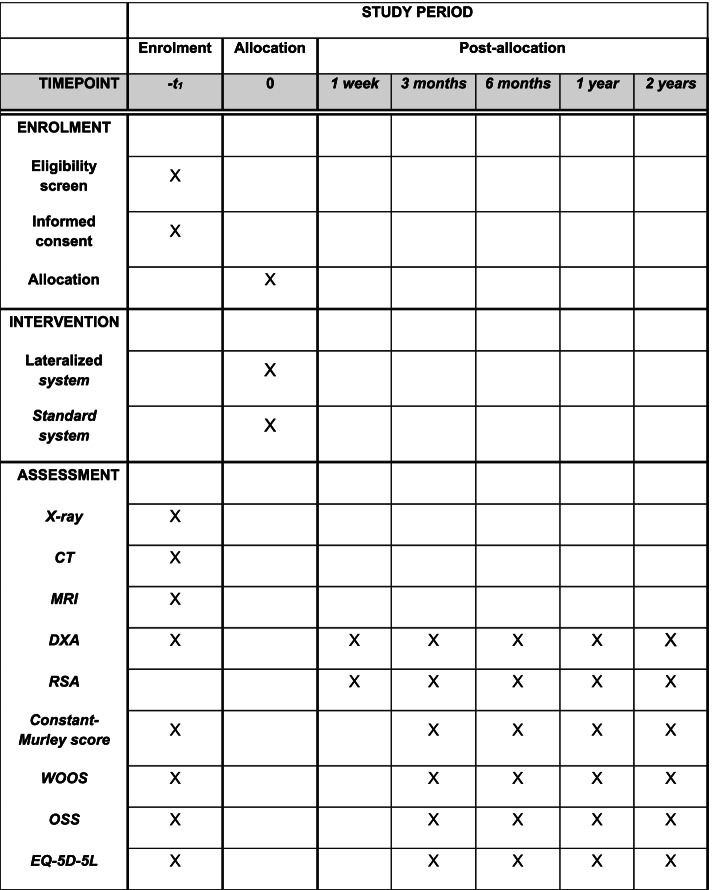
SPIRIT figure. Schedule of enrolment and assessments of the 56 radiostereometric analysis patients

**Fig. 4:**
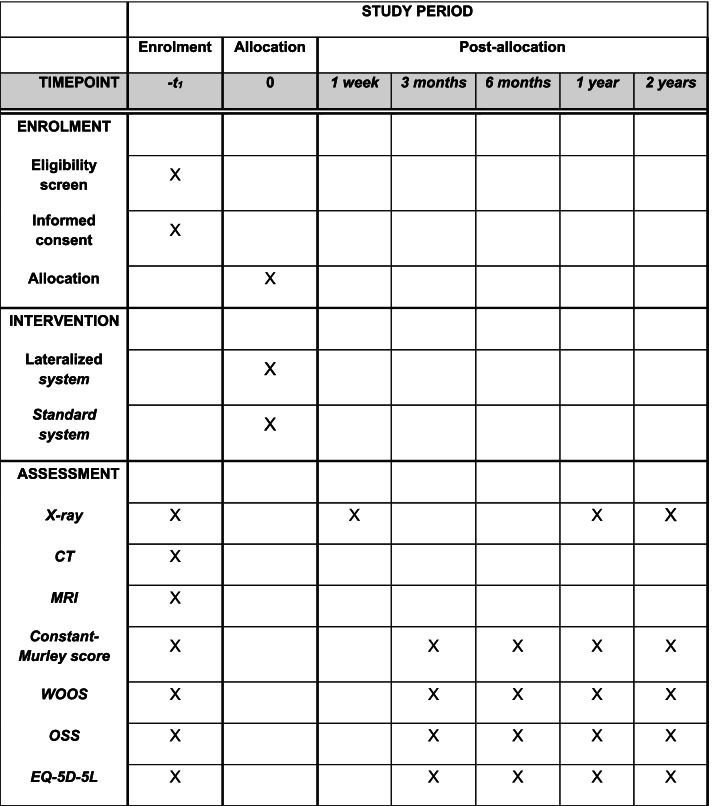
SPIRIT figure. Schedule of enrolment and assessments of the 66 patients who are not included in the radiostereometric analysis study

The patients will get an extra radiation dosage of 0.10 mSV including all the tests, which corresponds to the background radiation of 12 days in Denmark. In addition to the standard treatment the participating patients will have additional radiographic examinations including radiostereometric analysis, plain radiographs and DXA (Figs. 3 and 4).

### Randomization

After the assessment of the sample calculation, the study will aim towards including a total of 122 patients in which the first 56 patients will be examined with radiostereometric analysis and DXA. The patients are divided into two, equally sized and equally randomized to both arms, groups:

1. DELTA Xtend Reverse Shoulder System Lateralized Glenosphere Line Extension (intervention group)
2. Standard DELTA Xtend Reverse Shoulder System (control group).

In the operation room, the randomization is done on the trial laptop, short after the perioperative evaluation of the quality of bone stock. Thereby, the randomization is done when the patient is anaesthetized.

### Sequence generation and allocation

The randomization done on the trial laptop is done in the Research Electronic Data Capture (REdCap), in which the computer generates the randomization sequence by block randomization, stratified for gender and age and equally randomized to both shoulders (1:1 allocation). The primary investigator reads the allocation from the trial laptop to inform the surgeon. An independent statistician prepares the randomization table before the study is initiated.

### Blinding

It is not possible to blind the observer because the implant migration is assessed using radiostereometric analysis. However, patient-reported outcomes are solely assessed by the patients before the follow-up examination, without involving the surgeon or observer. The patients will be blinded to their allocated treatment the first 2 years after surgery.

The statistician, who performs the analysis, is blinded to the randomization.

### Surgical procedure

The procedures will be performed at Herlev and Gentofte Hospital, Copenhagen University Hospital. This unit performs around 300 primary and revision shoulder arthroplasties each year. From the evaluation of the number of surgeries from 2015 to 2016, it is expected to perform surgery on 75 patients with reverse shoulder arthroplasty for rotator cuff arthropathy every year. To minimize a potential learning curve and ensure a high surgical standard, the study is performed as a single-centre study and six experienced shoulder surgeons will be performing this type of surgery regularly using the same implant. To determine the potential learning curve applied by the lateralization devices, there will be done subgroup analyses at the end of the study, to compare the first and second half of the patients. Prior to the initiation of the study, each of the six surgeons shall have performed a minimum of 3 surgeries with the DELTA Xtend Reverse Shoulder System Lateralized Glenosphere Line Extension to minimize a potential learning curve problem.

The patient is under general anaesthesia and in a beach chair position during the procedure. Surgical technique, including soft tissue balancing and exposure instrumentation, is standardized for all patients. All surgeries are done with the standard deltopectoral approach and subscapularis tenodesis. All patients are treated with either (1) DELTA Xtend Reverse Shoulder System Lateralized Glenosphere Line Extension or (2) the standard DELTA Xtend Reverse Shoulder System according to the guidelines from the manufacturer. Perioperatively, approximately 20 tantalum markers (0.8 mm, Tilly Medical Products, Lund, Sweden) are placed in the bone at the proximal humerus and the glenoid around the components; we aim for the widest non-linear distribution, which will allow us to measure segmental motion with Mb-RSA software (Model-based RSA 4.1, 2003-2014 RSAcore Department of orthopedics Leiden University Medical Centre) using computer-aided design (CAD) models.

Size 1 and 2 humeral components will be used, in which all patients are treated with a cemented humeral monobloc component. Based on the medialization measured by plain radiographs, there will be used a 4-mm or 8-mm lateralized glenosphere if randomized for the intervention group. If randomized for the control group, a standard glenosphere is used. Prophylactic cloxacillin 2 g and benzylpenicillin 1.2 g are given preoperatively and at 6 and 12 h. If a patient cannot tolerate the standard prophylactic treatment, cefuroxime 1.5 g is given preoperative and at 6 and 12 h.

### Rehabilitation

All patients will receive a standard rehabilitation program led by a physiotherapist. A physiotherapist will one day postoperative instruct the patients in oedema prophylaxis. The patient will use a sling for 2 weeks, after which the patient has an appointment with the physiotherapist at the hospital for instructions in non-weight bearing training. Subsequently, the patient has an appointment with the physiotherapist once a week. There is a minimum of 3 months of training, longer if needed. Weight-bearing training is allowed after 6 weeks.

### Outcome measures

#### Primary radiological outcome

Migration of the glenoid components is assessed by model-based radiostereometric analysis, which is performed according to the guidelines of Valstar and Colleagues [12]. In this part of the study, the first 56 patients are included. Maximum total point motion (MTPM) after 2 years of follow-up is compared to the baseline value and is used as the primary radiostereometric analysis effect parameter. The accuracy of radiostereometric analysis will be assessed by at least 12 double examinations. The reference examination will be done within 1 week after the surgery, and the follow-up examinations are planned at 3 months, 6 months, 1 year and 2 years. Radiostereometric analysis is conducted using a uniplanar radiostereometric analysis arrangement (UmRSA®- Calibration Cage No 43 (hip, spine and shoulder)). The x-ray analysis is performed using the model-based radiostereometric analysis commercial software (RSAcore, Department of Orthopedics, Leiden University Medical Center, Leiden, The Netherlands), available at the Skaane University Hospital, Lund Sweden, where the analyses of radiostereometric analysis-x-rays will be conducted. The exact set-up for the radiostereometric analysis (e.g. various distances and the degree between the 2 x-ray tubes) will be specified from a small phantom study and measurements of the 6 pilot patients (3 with each of the 2 types of implants). 42 combined CAD models for MB-RSA will be delivered by Depuy Synthes (Raynham, MA, USA).

#### Primary functional outcome

*WOOS score*: The WOOS score is a disease-specific patient-reported outcome [18]. It contains 19 questions, divided into four groups: sports and work, physical symptoms, emotions, and lifestyle. The questions are answered using a visual analogue scale ranging from 0 to 100. The overall score is from 0 to 1900, where 1900 is the worst result. The scores are converted into a percentage of the maximum score to ease the interpretation. The difference in mean score between the preoperative score and the 2-year follow-up score is the primary value of interest. In this study, there is used a version of WOOS, which has been translated to Danish according to the guidelines of Guillemin, Bombardier and Beaton [19]. This version has been validated with classical test theory in a cohort of patients treated with shoulder arthroplasty for osteoarthritis [21].

#### Secondary radiological outcomes

*Plain radiographs*: the plain radiographs will be taken preoperatively, in the first week postoperatively, at 3 months, 1 year and 2 years. There will be used an anterior-posterior and lateral view. The radiographs will be used for evaluation of inferior scapular notching.

*DXA*: the first 56 patients will be included in this part of the study. DXA will be conducted preoperatively to evaluate BMD of the proximal humerus, scapula and distal forearm. BMD measurements of the distal forearm will be performed bilateral and used as reference to the shoulder and to adjust for changes in BMD that are not related to the shoulder arthroplasty. The difference in mean score between the preoperative BMD score and the 2-year follow-up score is the primary value of interest as it represents the change after the implant. The precision of DXA will be assessed by 12 double examinations.

#### Secondary functional outcomes

*Constant-Murley Score*: The Constant-Murley Score includes an evaluation of pain, range of motion, activities of daily living and strength. The maximum score for the evaluation of pain and activities of daily living is 35 points. For the objective evaluation, there is a maximum score of 65 points, of which 40 are from a range of motion and 25 are from strength. Thereby there is a collected maximum of 100 points, which indicates a shoulder without any disabilities. The difference in mean score between the preoperative score and the 2-year follow-up score is the primary value of interest. In this study, a Danish version [22] of the modified Constant score [23] is used.

*Oxford Shoulder Score (OSS)*: The OSS was invented as a measurement tool to assess function and pain after elective shoulder surgery [24]. It contains 12 questions with a score from 0 to 4. The total score ranges from 0 to 48, with 48 as the best score. The scores are converted into a percentage of the maximum score to ease the interpretation. The difference in mean score between the preoperative score and the 2-year follow-up score is the primary value of interest. In this study, the Danish version, which has been translated and validated with the classical test theory [25], of OSS is used.

*Complications and side effects*: Any incident of medical (cardiovascular, embolism, pneumonia) and surgical complications (nerve damage, fracture, infection, instability, disposition of component and dislocation) will be noted, as well as revision surgery (removal or exchange of any component).

*Pain and patient satisfaction*: Pain is evaluated on the day of examination with the VAS score with scores from 0 to 10, with 10 points as the worst possible pain. The patients will describe the outcome on a 7-point scale, with ‘much better’ as the best and ‘much worse’ as the worst. The difference in mean score between the preoperative score and the 2-year follow-up score is the primary value of interest.

*Economic assessment*: Quality-adjusted life years (QALY) is determined from the threshold of the acceptable cost-utility ratios for the health care system to use. In, respectively, Europe and England, the thresholds are set at 30,000 Euros and 20,000–30,000 pounds. The study will compare the cost-utility of the DELTA Xtend Reverse Shoulder System Lateralized Glenosphere Line Extension with the set thresholds and the cost-utility of the Standard DELTA Xtend Reverse Shoulder System. To estimate the QALY for each patient, there will be used EQ-5D-5 L. The difference in mean score between the preoperative score and the 2-year follow-up score is the primary value of interest. There will be defined a cost model from medical records, registries, data from patients and unit costs from the Danish health care system. Information regarding usage and readmission of pain medication, length of hospital stay and discharge destination will be noted.

### Follow-up

The follow-up time for included patients is 2 years (Figs. 3 and 4) with assessment within 1 week and at 3 months, 6 months, 1 and 2 years from the day of the surgery and thereby randomization. There will be conceived data on complications including revision surgery from medical records and hospital database after 10 years.

### Protocol violations, revision and drop-out

If the surgeon during the surgery regards it as impossible to insert a glenoid component, the surgery can be converted to a stemmed hemiarthroplasty using the DELTA Xtend humeral stem and a CTA head.

If patients drop out of the trial, it will be recorded along with the reason for drop-out. The patient in question will be included in the final mixed-effect model analysis, except for patients who drop out before 3 months, who cannot be included in the analysis. At the end of the study, a sensitivity analysis will be performed to evaluate the impact of missing data on the overall results of the trial. It will be recorded if there are indications that some patients do not comply with rehabilitation.

In case of revision surgery, reason and new arthroplasty type will be recorded. Additionally, the patient will prior to revision surgery be assessed by DXA, radiostereometric analysis, patient-reported outcome and clinical outcome if possible. The patient will stay included in the study and the 2-year analysis with the latest follow-up.

### Modification of the protocol

Any protocol modifications which might have an impact of the study will immediately be reported to all investigators and trial registries.

### Statistics

Prior to the analysis, the nature of the data is assessed. It is expected to analyse the changes between pre- and postoperative assessment with paired parametric statistics. The differences between the groups are expected to be analysed using parametric statistics (Student *t*-test). The radiostereometric analysis data is not expected to be normally distributed and will be analysed using non-parametric analysis of variance over time. The differences between these groups will be analysed with Mann-Whitney *U* test. Because of the nature of repeated measures in this study, it is expected to use a mixed effects model to analyse the overall results. Data on radiostereometric analysis and DXA will be analysed at the end of follow-up for the first 56 patients. No other interim data-analysis will be carried out.

### Sample size calculation radiostereometric analysis

Sample size calculation for radiostereometric analysis (maximum total point motion of the glenoid component after 2 years will be used as primary radiostereometric analysis effect parameter) was performed with an expected standard deviation (SD) of 0.4 mm, a minimally clinically important difference of 0.4 mm, a significance level at 5% and power of 0.90 and resulted in 22 participants in each group (56 participants with allowance for 20% drop-out) [26]. There are no data metrics for migration of the glenoid component in reverse shoulder arthroplasty, but the sample size fits with estimates in previous studies comparing two different orthopaedic implants. The argument is that RSA has high accuracy and, therefore, that few patients are needed [12].

### Sample size calculation WOOS

The standard deviation of WOOS is set at 15 (15% of a maximum score). The minimally clinically important difference is set at 10 (10% of a maximum score), the significance level is set at 5% and the power of 0.90. With these assumptions, 48 patients are needed in each group resulting in a study population with 122 participants [26] (allowing for 20% drop-out). MCID of WOOS for patients with reverse shoulder arthroplasty for degeneration of the glenohumeral joint with either rotator cuff insufficiency or severe posterior glenoid wear has not been defined. Thus, the estimates used in the sample size calculation were based on studies which included patients with an anatomical total shoulder arthroplasty for osteoarthritis [27, 28].

## Discussion

This study aims to determine if DELTA Xtend Reverse Shoulder System Lateralized Glenosphere Line Extension has a better outcome than the Standard DELTA Xtend Reverse Shoulder System. The patients will be divided into two groups and equally randomized and treated. There is a possibility of being treated with what turns out to be an inferior arthroplasty. Today, it is not known if one treatment is better than the other as the two types of glenospheres are considered equal effective and safe.

Sirveaux et al. [4] reported that inferior scapular notching was associated with increased risk of revision, and Mollon et al. [5] described lower clinical outcomes for these patients. This study aims to explore metallic lateralization as a solution to prevent inferior scapular notching and subsequently better outcomes. Lateralization of the glenoid off-set has earlier been reported as the most efficient technique to avoid inferior scapular notching [8]. Furthermore, lateralization has been described to improve movement [9] but at the same time makes greater demands to the deltoid muscle [29]. There has also been reported a higher rate of loosening [8]. Implant migration can be the first sign of loosening. Radiostereometric analysis has been used to measure implant migration in numerous studies for more than 20 years [30], but this is, to our knowledge, the first time radiostereometric analysis is used to compare the outcome for conventional reverse shoulder arthroplasty and lateralized reverse shoulder arthroplasty in a randomized controlled trial. DXA has been used in shoulder patients before.

The model-based radiostereometric analysis technique is less precise than the marker-based method. However, precision error values are still acceptable for clinical studies aimed at evaluating implant migration [31]. The use of radiostereometric analysis for the evaluation of implant migration has been used frequently in the evaluation of hip and knee arthroplasty surgery. It has been shown that the late revision due to aseptic loosening of the tibial component in total knee arthroplasty is consistent with early radiostereometric analysis findings of continuous migration past the first postoperative year [32]. Therefore, it has been suggested that a small series of new arthroplasties should be monitored with radiostereometric analysis the first 2 years postoperatively, as part of a safe phased introduction of new arthroplasties [30, 33, 34].

The standard follow-up time after a shoulder arthroplasty at our department is 3 months. The patients included in this study will additionally be examined within 1 week and at 6, 12 and 24 months. This can both contribute to the patients feeling more secure by handling problems as they come or entail a long-lasting feeling of being ill and furthermore being time-consuming. The patients will not be financially compensated for the extra visits.

As part of safe implementation of a new orthopaedic surgical product [35], after being tested by the manufacturer, this study will test the lateralized glenosphere with radiostereometric analysis and a RCT study. If it finds an advantage without infection as an expense, it can be implemented in the treatment of patients without further testing. The treatment will be controlled through the Danish Shoulder Arthroplasty Registry [36, 37].

The study aims to earn knowledge of the treatment option with the most optimal outcome for future patients needing a reverse shoulder arthroplasty.

### Trial status

Version 1.1, May 18, 2022

Start inclusion: August 1, 2022

Finish date of recruitment to the first part of the study: July 31, 2023

Finish date of recruitment of all 122 patients: July 31, 2024

Finish date of follow-up for the first part of the study: July 31, 2025

Finish date of follow-up for all 122 patients: July 31, 2026

## Supplementary Information

**Additional file 1: Appendix 1.** SPIRIT Checklist for Trials

**Additional file 2: Appendix 2.** Deltagerinformation

**Additional file 3: Appendix 3.** Deltagerinformation

**Additional file 4: Appendix 4.** Informed consent

**Additional file 5.**

## Data Availability

The electronic data will be stored in closed drives and printed data will be stored in closed cabinets. The datasets used and/or analysed during the current study are available from the corresponding authors on reasonable request. The study has been notified to Pactius and has approval number P-2021-231.

## Abbreviations

ROM: Range of motion
PROM: Passive range of motion
ASA: American Society of Anaesthesiology
OSS: Oxford Shoulder Score
WOOS score: Western Ontario Osteoarthritis of the Shoulder index
VAS score: Visual analogue scale
DXA: Duel energy x-ray absorptiometry
BMD: Bone mineral density
REdCap: Research Electronic data Capture
MB-RSA: Model-based radiostereometric analysis
CAD models: Computer-aided design models
MTPM: Maximum total point motion
QALY: Quality-adjusted life years
SD: Standard deviation
