# Supplementary material for: Implant migration and functional outcome of Reverse Shoulder Lateralized Glenosphere Line Extension System: a study protocol for a randomized controlled trial
Source: Trials. 2022 Jul 19;23:579. doi: 10.1186/s13063-022-06482-8 (PMC9295266; doi:10.1186/s13063-022-06482-8)
Supplement: Supplementary file 3 — Additional file 3: Appendix 3. Deltagerinformation [file 13063_2022_6482_MOESM3_ESM.pdf]

Deltagerinformation

Migration and functional outcome of DELTA Xtend Reverse Shoulder Lateralized Glenosphere Line Extension System – A Randomized Controlled Trial

Vi vil spørge, om du vil deltage i et videnskabeligt forsøg, der udføres på Afdeling for Led- og Knoglekirurgi, Gentofte Hospital.

Det er frivilligt at deltage i forsøget. Du kan når som helst og uden at give en grund trække dit samtykke tilbage. Det vil ikke få konsekvens for din videre behandling.

Du har ret til at medbringe en bisidder ved første samtale og har ret til minimum 24 timers betænkningstid.

Formål med forsøget:
Ved svære tilfælde af rotator-cuff artropati i skulderen (defineret som degeneration af skulderleddet og slid eller skade af mindst en rotator-cuff sene) eller slidforandringer i skulderleddet med betydeligt knogletab kan det være nødvendigt at indsætte en revers skulderprotese. Aktuelt anvendes der som udgangspunkt en standard ledkomponent ved denne protesetype. Dette har medført gode resultater, men også en række komplikationer der relaterer sig til protesens omdrejningspunkt. Ved at anvende en ledkomponent, der ændrer omdrejningspunktet for protesen vil man formodentlig kunne reducere disse komplikationer. Dette er dog ikke undersøgt til bunds endnu, hvorfor det er formålet med dette studie at undersøge denne hypotese og dermed i fremtiden kunne tilbyde den bedst mulige behandling.

Plan for forsøget:
Vi vil undersøge ovenstående formål via et studie, hvor vi sammenligner resultaterne mellem patienter, der er blevet opereret med henholdsvis en skulderprotese med en standard ledkomponent og patienter opereret med en skulderprotese med en komponent med ændret omdrejningspunkt. Vi har brug for i alt 122 patienter til forsøget.

Der vil for hver patient, der medvirker i forsøget blive trukket lod om hvilken protesetype, som patienten skal opereres med. Patienten vil ikke selv være vidende om hvilken protese han/hun bliver opereret med.

I forbindelse med forsøget, skal man møde i alt 7 gange. Første gang er før operationen, anden gang er i forbindelse med selve indlæggelsen og operationen og de sidste 5 gange er henholdsvis 1 uge, 3 måneder, 6 måneder og 1 og 2 år efter operationen. Normalt i forbindelse med operation med indsættelse af en skulderprotese skal man møde 3 gange, nemlig før operationen, i forbindelse med indlæggelsen og operationen og efter 3 måneder.

De enkelte besøg vil inkludere følgende undersøgelser:

- Før operationen: Røntgen, CT, MR, spørgeskemaer, undersøgelse af skulderfunktion
- Efter 1 uge: Røntgen
- Efter 3 måneder: Spørgeskemaer, undersøgelse af skulderfunktion
- Efter 6 måneder: Spørgeskemaer, undersøgelse af skulderfunktion
- Efter 1 år: Røntgen, spørgeskemaer, undersøgelse af skulderfunktion
- Efter 2 år: Røntgen, spørgeskemaer, undersøgelse af skulderfunktion

Bivirkninger, risici, komplikationer og ulemper:
Risikoen ved at indgå i dette forsøg vurderes at være minimal. Aktuelt anses de to protesetyper for at være ligeværdige. Dog vil der selvsagt være en risiko for, at den ene forsøgsgruppe ved senere analyser viser sig at være blevet behandlet med en protese med dårligere resultater end den anden.

Man vil som deltager i forsøget skulle komme til 4 besøg ud over standardregimet. Dette er selvsagt tidsforbrugende og kan derfor ses som en ulempe. Men på den anden side vil det også medføre, at eventuelle usikkerheder eller problemer kan løses hurtigere og lettere.

Scanningerne og røntgenundersøgelserne vil samlet medføre en stråledosis på 0,10 mSv. Dette giver en samlet risiko for stokastisk skade i størrelsesorden 1 til 100.000.

Der kan være risici og belastninger ved forsøget, som vi ikke på forhånd kender til. Vi beder dig derfor om at fortælle, hvis du oplever uforudsete problemer, mens forsøget står på. Hvis vi opdager problemer, som vi ikke allerede har fortalt dig om, vil du naturligvis blive orienteret med det samme, og du vil skulle tage stilling til, om du ønsker at fortsætte i forsøget.

Standardbehandling:
Patienter som tilbydes operation med en omvendt skulderprotese, er indtil nu blevet opereret med en skulderprotese med en standard ledkomponent. Ønsker du derfor ikke at deltage i forsøget, vil du blive tilbudt vores standardbehandling med denne protesetype og du vil følge det vanlige regime med kun en opfølgning efter 3 måneder.

Journaloplysninger:
I forbindelse med forsøget anvendes der oplysninger fra din patientjournal. De oplysninger vi er interesserede i, er om du lider af kroniske sygdomme, hvilken medicin du tager til daglig, uddannelse, beskæftigelse, tobaks- og alkoholvaner, alder samt køn. Oplysningerne bruges kun i forbindelse med dette forsøg og videregives ikke til andre. Ved samtykke til deltagelse i forsøget giver du den forsøgsansvarlige og dennes repræsentanter adgang til de relevante journaloplysninger for at kunne gennemføre, overvåge og kontrollere forsøget.

Oplysningerne om dig og din skulder vil blive opbevaret i en sikret database. Dit CPR-nr. vil blive fjernet fra denne database efter 10 år. De personer der vil have adgang til denne database i denne periode, er kun fagpersonale med tilknytning til forsøget og har alle tavshedspligt.

Behandling af personoplysninger:
Der vil i forsøget blive behandlet personoplysninger. Dette gøres med overholdelse af databeskyttelsesloven og databeskyttelsesforordningen.

Forsøgets nytte:
Forsøget vil give os ny viden om den bedst mulige behandling af rotator-cuff artropati og slidforandringer i skulderleddet med betydeligt knogletab, til gavn for fremtidige patienter. Da det er uvist om den ene protese er bedre end den anden, er der både en risiko for at blive behandlet med en dårligere protese og en chance for at blive behandlet med en bedre protese. Desuden er der for dig den nytte, at du vil blive grundigere undersøgt end ved en vanlig operation.

Udelukkelse fra og afbrydelse af forsøg:
Såfremt det under operationen viser sig at din knoglekvalitet er insufficient, vil du udgå af forsøget.

Forsøget afbrydes før tid, hvis det under databehandlingen fremgår, at der er en markant forskel i resultaterne mellem de to grupper.

Økonomi:
Det er de forsøgsansvarlige forskere på Afdeling for Led- og Knoglekirurgi, Gentofte Hospital, som har taget initiativ til forsøget. Studiet er efterfølgende blevet bevilget støtte med et beløb på 1.722.099 DKK fra protesefirmaet Depuy Synthes. Pengene vil gå til både udarbejdelse af forsøget, samt løn til ph.d.-studerende. Den forsøgsansvarlige eller øvrige forsøgsdeltagere har ingen tilknytning til støttegiver.
Den forsøgsansvarlige er tilknyttet Herlev og Gentofte Hospital i og med, at projektet udarbejdes herfra.

Vi håber, at du med denne information har fået tilstrækkeligt indblik i, hvad det vil sige at deltage i forsøget, og at du føler dig rustet til at tage beslutningen om din eventuelle deltagelse. Vi beder dig også om at læse det vedlagte materiale "Forsøgspersonens rettigheder i et sundhedsvidenskabeligt forskningsprojekt".

Hvis du vil vide mere om forsøget, er du meget velkommen til at kontakte en af nedenstående:

Forsøgsansvarlig

Jeppe Vejlgaard Rasmussen, læge, Ph.d.
Afdeling for Led- og Knoglekirurgi
Herlev og Gentofte Hospital
Telefon: +45 40561859
Mail: jeppe.vejlgaard.rasmussen@regionh.dk

Kontaktperson

Marie Louise Jensen, læge
Afdeling for Led- og Knoglekirurgi
Herlev og Gentofte Hospital
Telefon: +45 25717963
Mail: marie.louise.jensen.02@regionh.dk

Version 1.0, 01.07.2021
