# Supplementary material for: Implant migration and functional outcome of Reverse Shoulder Lateralized Glenosphere Line Extension System: a study protocol for a randomized controlled trial
Source: Trials. 2022 Jul 19;23:579. doi: 10.1186/s13063-022-06482-8 (PMC9295266; doi:10.1186/s13063-022-06482-8)
Supplement: Supplementary file 4 — Additional file 4: Appendix 4. Informed consent [file 13063_2022_6482_MOESM4_ESM.pdf]

Informeret samtykke til deltagelse i det sundhedsvidenskabelige forskningsprojekt:

Migration and functional outcome of DELTA Xtend
Reverse Shoulder Lateralized Glenosphere Line Extension System

Version 1, 24.06.2021

Erklæring fra forsøgspersonen:

Jeg har fået skriftlig og mundtlig information og jeg ved nok om formål, metode, fordele og ulemper til at sige ja til at deltage.

Jeg ved, at det er frivilligt at deltage, og at jeg altid kan trække mit samtykke tilbage uden at miste mine nuværende eller fremtidige rettigheder til behandling.

Jeg giver samtykke til at deltage i forskningsprojektet og har fået en kopi af dette samtykkeark, samt en kopi af den skriftlige information om projektet til eget brug.

Forsøgspersonens navn: ____

Dato: ____________________ Underskrift: ____

Erklæring fra den der afgiver information:

Jeg erklærer, at forsøgspersonen har modtaget mundtlig og skriftlig information om forsøget.

Efter min overbevisning er der givet tilstrækkelig information til, at der kan træffes beslutning om deltagelse i forsøget.

Navnet på den, der har afgivet information: ________________________________

Dato: ____________________ Underskrift: ____
