# Supplementary material for: Implant migration and functional outcome of Reverse Shoulder Lateralized Glenosphere Line Extension System: a study protocol for a randomized controlled trial
Source: Trials. 2022 Jul 19;23:579. doi: 10.1186/s13063-022-06482-8 (PMC9295266; doi:10.1186/s13063-022-06482-8)
Supplement: Supplementary file 5 — Additional file 5. [file 13063_2022_6482_MOESM5_ESM.pdf]

**Contact information for funding contact:**

Jamie McDonald, Staff Clinical Research Scientist, Investigator Initiated Studies and External Research.

Depuy Synthes, a Johnson & Johnson company

St. Anthony's Road

Beeston

Leeds

West Yorkshire

United Kingdom

LS11 8DT

Tel: +44 (0) 7500 577 545

e-mail: jmcdon10@its.jnj.com

**Contact information for external assessor**

Name: Kristoffer W. Barfod, PhD, MC, clinical lector

Position: Chief physician at Arthroscopic Center, Hvidovre Hospital

E-mail: kbarfod@dadlnet.dk

Tel: +45 40134773
